# The novel and powerful ICD-11 classification system for neoplasm coding: a comparative study with the ICD-O

**DOI:** 10.1186/s12911-022-02077-0

**Published:** 2022-12-16

**Authors:** Yicong Xu, Jingya Zhou, Yi Wang

**Affiliations:** 1 Medical Records Room, Department of Medical Administration, the Second Affiliated Hospital, Zhejiang University School of Medicine, Hangzhou, 310009 Zhejiang China; 2 Department of Medical Records, Peking Union Medical College Hospital, Chinese Academy of Medical Sciences and Peking Union Medical College, Beijing, 100730 China; 3 Collaborating Center for the WHO Family of International Classifications in China, Beijing, 100730 China

**Keywords:** ICD-11, ICD-O, International classification of diseases, Neoplasm coding, Differences, Compatibility

## Abstract

**Background:**

The International Classification of Diseases (ICD) and the International Classification of Diseases for Oncology (ICD-O) are both widely used global classification systems. In 2018, the initial release of the ICD-11 was published by the World Health Organization (WHO), integrating the morphology section of the ICD-O.

**Methods:**

This paper aims to provide potential ICD-11 users with a profound understanding of the neoplasm classifications of the ICD-11 by analysing the differences and relationships between the ICD-11 and ICD-O in terms of the coding framework, compatibility and intelligence level.

**Results:**

The ICD-11 and ICD-O have remarkable differences in coding structure. Compared to the ICD-O, the ICD-11 has the following advantages: adding histopathology to the stem codes, obtaining a meaningful minimum amount of information through stem codes for statistics, supporting the usage of ICD-O morphology categories and capturing clinical details via extension codes for multiaxial coding. In addition, the rich Foundation Component, linearization derived from the Foundation Component and updating mechanism all support the compatibility of the ICD-11 with other classification systems. Notably, the WHO provides terminology coding with a smart coding tool, and coding in the ICD-11 can draw on statistical codes and uniform resource identifiers (URIs) simultaneously.

**Conclusions:**

The ICD-11 represents a novel classification system with distinguishing features that include facilitating statistics, multiaxial coding, coding granularity, compatibility and intelligence. These features enable the ICD-11 to be more powerful for neoplasm coding than the ICD-O and basically meet the needs of stakeholders.

## Background

The International Classification of Diseases (ICD) and the International Classification of Diseases for Oncology (ICD-O) have been jointly used and developed for nearly fifty years, and both are widely used global classification systems. Two editions of the ICD-O, the ICD-O-1 and the ICD-O-2, were published by the World Health Organization (WHO) in 1976 and 1990, respectively, and they were usually used in conjunction with Chapter II, Neoplasms, of the ICD-9 and ICD-10. In 2000, the WHO released the third edition of the ICD-O (ICD-O-3). The site section of this third edition remains the same as in the second edition, while the morphology section has been revised [1]. In April 2019, the WHO published the latest version of the ICD-O-3.2, which is more suitable for current medical knowledge, and the International Agency for Research on Cancer (IARC) assisted the WHO in the revision.

In addition, the ICD-10 was also revised, and the initial release of the ICD-11 was published in 2018. Although a stable version of the ICD-11 for preparation of implementation was released by the WHO, official adoption by all WHO member states for mandatory use starting in January 2022 for recording and reporting causes of illness and death occurred in May 2019. A design principle for the ICD-11 was to maintain good backwards compatibility with the ICD-10, so the overall framing of diseases in the ICD-11 for Mortality and Morbidity Statistics (ICD-11 MMS) remains similar to that in the ICD-10, which is reflected in the similar titles and sequence of chapters [2]. However, there are many changes at specific levels, especially the classification hierarchy and classification axis of Chapter II (Neoplasms), as well as changes in the coding structure and integrating the morphology section of the ICD-O. Nevertheless, these changes could lead to obstacles for users.

Currently, the ICD-11 is not fully understood, although 35 countries around the world are using it [3]. The WHO is committed to supporting all countries as they move towards implementing and scaling up the use of the ICD-11 [4]. In January 2022, the National Health Commission of the People's Republic of China launched a pilot application program to promote the ICD-11, with fifty-nine large general public hospitals from all provinces participating. The program is devoted to identifying key and difficult problems in the usage of the ICD-11, providing a practical basis for the smooth transition to the ICD-11 and continuously improving its applicability. Therefore, it is essential for potential users who are accustomed to using the ICD-O to better understand the differences and relationships between the ICD-O and ICD-11. This paper compares and analyses the differences and relationships between the ICD-11 and ICD-O in terms of the coding framework, compatibility and intelligence level. We hope that this paper will make it easier for users to transition to the ICD-11 and provides a reference for the development of data acquisition, processing and analysis tools in the future.

## Coding framework

### Comparison of the coding structure

A complete ICD-O code requires 10 digits or characters to identify the topographic site (4 characters), histological type (4 digits), behaviour (1 digit), and grade or differentiation of a neoplasm or its equivalent in leukaemias and lymphomas (1 digit) [1], which can be divided into two parts (topography code, morphology code). The topography code is based on the malignant neoplasm section of the ICD-10, ranging from C00 to C80, and it remains the same for all types of neoplasms at the same site.

In contrast, the ICD-11 breaks the tradition of separated coding of topography and morphology, introducing new terms for stem codes, extension codes, precoordination, postcoordination and cluster codes [5]. Some stem codes contain topography and histopathology in a precombined fashion, which is referred to as ‘precoordination’. Extension codes provide more specific information (e.g., laterality, anatomy, histopathology, stages, grading) linked to stem codes, which is referred to as ‘postcoordination’.

Stem codes are generally six-digit codes, and the coding structure is E_1_D_2_1_3_E_4_.E_5_E_6_. The first character of the code relates to the chapter number, so the code of Chapter II (Neoplasms) starts with number ‘2’. The second and third characters of the code are a letter and number, respectively, while the fourth character of the code is a letter or number. The codes of Chapter II range from 2A00 to 2F9Z.

Extension codes are also basically six-digit codes composed of letters and numbers, starting with the letter ‘X’, which are generated randomly by a computer. Stem codes can stand alone, and yet extension codes cannot be used independently except for adding detail to stem codes. Stem codes and extension codes are connected by an ampersand (&), while two stem codes are connected by a forward slash (/), and these coding groups are called cluster codes.

For example, ‘well-differentiated invasive ductal carcinoma in stage 1 of the breast in the left upper outer quadrant’ would be coded 2C61.0&XK8G&XA2Q54&XS56&XS1G by the ICD-11; the meaning of each code is shown in Table 1. The ICD-O counterpart is C50.4 (upper outer quadrant of the breast), 8500/31 (infiltrating duct carcinoma, well-differentiated).

**Table 1:** Coding structure of the ICD-O and ICD-11

Neoplasm	ICD-O-3.2		ICD-11	
	Topography code	Morphology code	Stem code	Extension code
Well-differentiated invasive ductal carcinoma in stage 1 of the breast in the left upper outer quadrant	C50.4	8500/3^a^1^b^	2C61.0	&XK8G&XA2Q54&XS56&XS1G

In the ICD-11, 2C61.0 represents invasive ductal carcinoma of the breast; XK8G represents left; XA2Q54 represents the upper outer quadrant of the breast; XS56 represents grade I; XS1G represents stage I

The ICD-11 coding tool website: https://icd.who.int/devct11/icd11_mms/en/current (2022–10-25)

^a^The behaviour code includes 0–3, 6 and 9 corresponding to benign, uncertain whether benign or malignant, in situ, primary site malignant, metastatic site malignant, and uncertain whether primary or metastatic site malignant, respectively

^b^The grading codes 1 to 4 are used to designate grades I to IV, respectively

Overall, the coding structure of the ICD-11 is completely different from that of the ICD-O.

### Function of the stem codes and extension codes

The stem codes were designed to collect a meaningful minimum amount of information through only one code per case in use cases, and adding histopathology to the stem codes is one of the breakthrough changes in the ICD-11. They not only facilitate the statistical analysis of common and important histopathology neoplasms but also much better conform to the expression habits of clinical neoplasm terms.

The addition of Chapter X, called ‘Extension Codes’, which contains multidimensional information on concepts including laterality, anatomy, histopathology, stage and grade details, supports detailed clinical abstraction and allows for multiaxial coding. Through postcoordination, extension codes have the potential to improve coding granularity [6], which has been proven by many studies [7–9]. In addition, its flexibility should not be ignored [10].

As shown in Table 1, the stem code (2C61.0) describes the site and histopathology, and the optional usable extension codes provide the rest of laterality, detailed anatomy, stage and grading information, while the ICD-O does not provide laterality and stage information. As the smallest unit containing the most meaningful information, stem codes are convenient for statistics, different from the additional optional extension codes for clinical abstraction.

Stem codes provide specificity to the ICD-11. First, the ICD-11 clearly distinguishes histopathology via the stem codes, such as ‘adenocarcinoma of appendix’ (2B81.0) and ‘mucinous adenocarcinoma of appendix’ (2B81.1), as shown in Table 2. However, when applying the ICD-O, all cases would be coded as C18.1 (appendix), and they are only distinguished via morphology codes. Second, the ICD-11 clearly distinguishes behaviour via the stem codes, such as neoplasms of uncertain behaviour (2F70-2F7Z) and unknown behaviour (2F90-2F9Z).

**Table 2:** Examples of distinguishing histopathology via stem codes

Neoplasm	ICD-O-3.2		ICD-11	
	Topography code	Morphology code	Stem code	Extension code
Adenocarcinoma of appendix	C18.1	8140/3	2B81.0	
Mucinous adenocarcinoma of appendix	C18.1	8480/3	2B81.1	
Tubular adenoma of appendix	C18.1	8211/0	2E92.4Y	&XA8PW4&XH7SY6

In the ICD11, XA8PW4 represents  appendix; XH7SY6 represents  tubular adenoma. The ICD-11 coding tool website: https://icd.who.int/devct11/icd11_mms/en/current (2022-6-24)

The extension codes also bring specificity to the ICD-11, especially if one or more extension code can be linked when coding a specific condition. For example, when a single tumour overlaps the boundaries of two or more categories or subcategories and its point of origin cannot be determined [1], the ICD-O generally uses subcategory ‘.8’, such as C16.8 (overlapping lesion of the stomach). However, this rule does not apply in the ICD-11, which rarely provides overlapping codes directly. Multiple extension codes (&XA7UE1&XA56K7 or &XA6P89&XA56K7) could fully represent the overlapping sites, as shown in Table 3.

**Table 3:** Examples of different expressions for a tumour overlapping the boundaries according to the ICD-O-3.2 and ICD-11 MMS

Neoplasm	ICD-O-3.2		ICD-11	
	Topography code	Morphology code	Stem code	Extension code
Adenocarcinoma of the gastric corpus and fundus	C16.8	8140/3	2B72.0	&XA7UE1&XA56K7
Adenocarcinoma of the gastric pylorus and fundus	C16.8	8140/3	2B72.0	&XA6P89&XA56K7

The ICD-11 coding tool website: https://icd.who.int/devct11/icd11_mms/en/current (2022-6-28).

### Morphology categories

As one of the most important extension codes, the morphology categories in the ICD-11 and ICD-O are basically the same. Referring to the ICD-O-3.2 found at the International Association of Cancer Registries (IACR) website [11], the terms are divided into three levels according to the hierarchy. The first level of classification (first 3 digits) corresponds to the morphological description of the neoplasm. Among them, only the ‘Hodgkin and non-Hodgkin lymphoma’ category and ‘Leukaemia’ category have the second-level classification, while the others do not. Finally, the third level of classification (five-digit code) corresponds to the most detailed category, a total of 2894 terms (see Table 4). The same neoplasm can be referred to in different ways; for example, 8240/3 can be verbalized as a carcinoid or neuroendocrine tumour (NET) [12]. To cope with this variation, the ICD-O-3.2 documents this variation marking as preferred when a given verbalization is preferred or more standard than the others, and the others are marked as synonyms. Therefore, the terms listed under the same code number are marked with preferred, synonym and related. The purpose of the synonyms is to help registrars find the correct morphology code, even if a term other than the preferred one has been used [12].

**Table 4:** Examples of the three levels of categories in the ICD-O-3.2

First-level category		Second-level category	Most detailed category
Code range	Term		
800	Neoplasms, NOS		Neoplasm, benign^a^ (8000/0)
			Neoplasm, malignant^a^ (8000/3)
			Neoplasm, metastatic^a^ (8000/6)
814–838	Adenomas and adenocarcinomas		Neuroendocrine tumour, NOS^a^(8240/3)
			Carcinoid, NOS^b^(8240/3)
			Bronchial adenoma, carcinoid^c^(8240/3)
959–972	Hodgkin and non-Hodgkin lymphomas	Hodgkin lymphomas (965–966)	Hodgkin lymphoma, NOS^a^(9650/3)
			Hodgkin granuloma^a^ (9661/3)
980–994	Leukaemias	Lymphoid leukaemias (981–983)	Lymphoid leukaemia, NOS^a^(9820/3)
			Prolymphocytic leukaemia, B-cell type^a^ (9833/3)

^a^Preferred

^b^Synonym

^c^Related

According to the first-level category, morphology is divided into forty-nine categories in the ICD-O-3.2, ranging from 8000/0 to 9993/3. The histopathology provided by the ICD-11 MMS [13] is divided into forty-eight categories, removing the category ‘Neoplasms, NOS’ (800), which is set as a grey entity and has no code on the website.

The category ‘Neoplasms, NOS’ is a residual category in the ICD-O, so it should be avoided except for cases that are clinically diagnosed but not confirmed by pathology. In contrast, the ICD-11 replaced functions of the category ‘Neoplasms, NOS’ by changing its grouping structure. The previous ICD-10 group Neoplasms of uncertain or unknown behaviour has been split into two separate groups—Neoplasms of uncertain behaviour and Neoplasms of unknown behaviour, and the unknown behaviour group does not need to express morphological types. The almost identical morphological categories indicate the compatibility of the ICD-11 and ICD-O.

## Backwards and forwards compatibility

The ICD-11 has good backwards compatibility, which is completely reflected in the rich Foundation Component. The Foundation Component, referring to a database, has approximately 80,000 entries and 40,000 synonyms [2], each characterizing a disease, injury, syndrome, external cause and so on. Not only have the anatomical subdivisions of the ICD-10 and ICD-O at the 3-character level [14] been preserved in the ICD-11 but also the TNM classification and histopathology from the ICD-O for tumour description have been integrated.

**Fig. 1:**
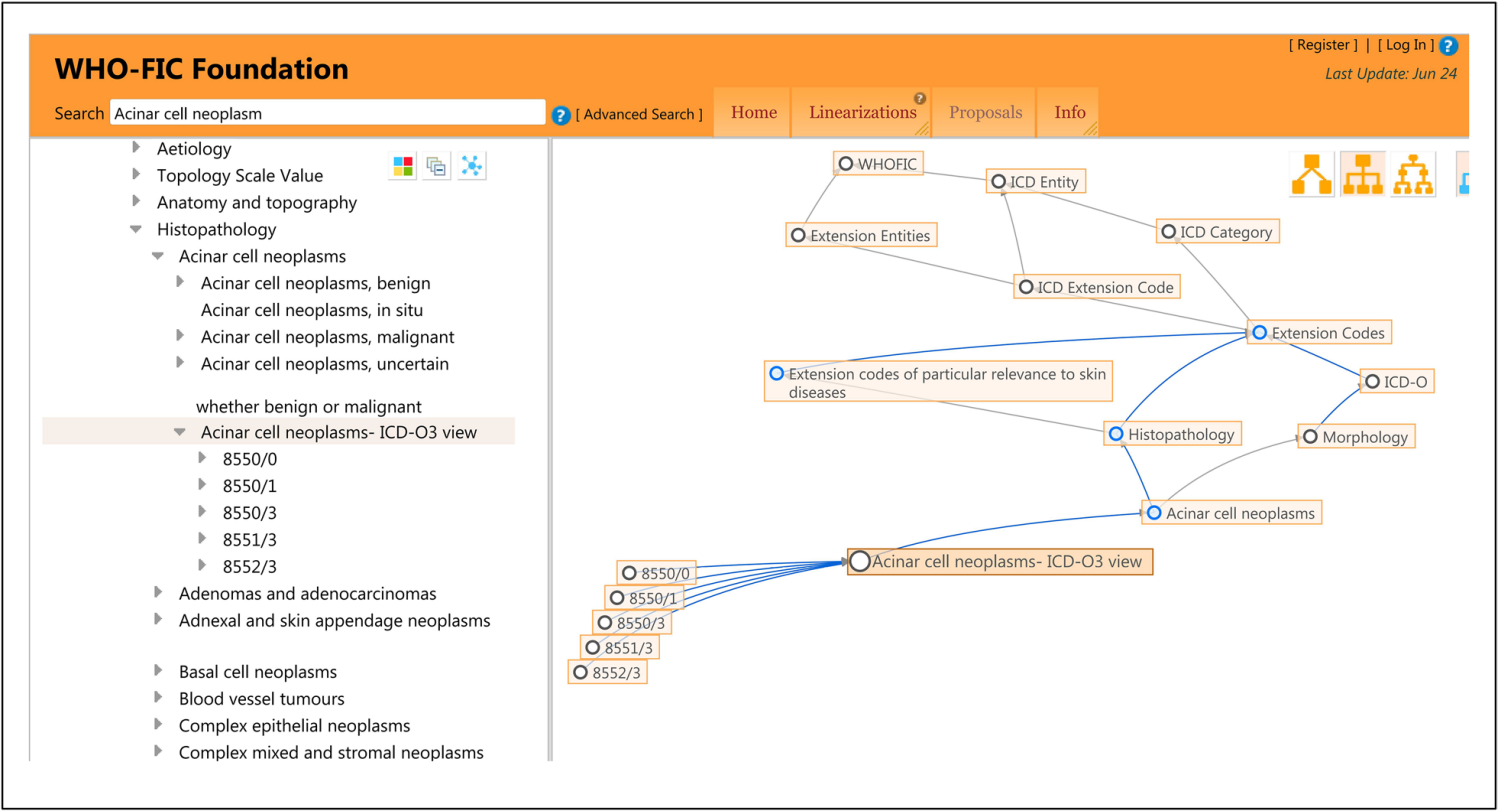
An example of linearization within the Foundation Component by the ICD-11 (2022/6/25). ‘Acinar cell neoplasms’ has two parents, namely, ‘Histopathology’ and ‘Morphology’. ‘Acinar cell neoplasms’ also has two grandfathers, namely, ‘extension codes’ and ‘ICD-O’. In addition, ‘extension codes’ is the parent of ‘ICD-O’

An ICD-O view is integrated within the Foundation Component, from which the morphology content of the ICD-O is fully reflected in the histopathology part of the ICD-11. For example, Fig. 1 shows the relationship between the Foundation Component and linearization as well as the relationship between the histopathology of the ICD-11 and the morphology of the ICD-O for ‘Acinar cell neoplasms’. Obviously, the ICD-11 integrates the morphology section of the ICD-O and ICD-O linearization. Hence, accessing the information of the ICD-O from Chapter II of the ICD-11 is more convenient.

**Fig. 2:**
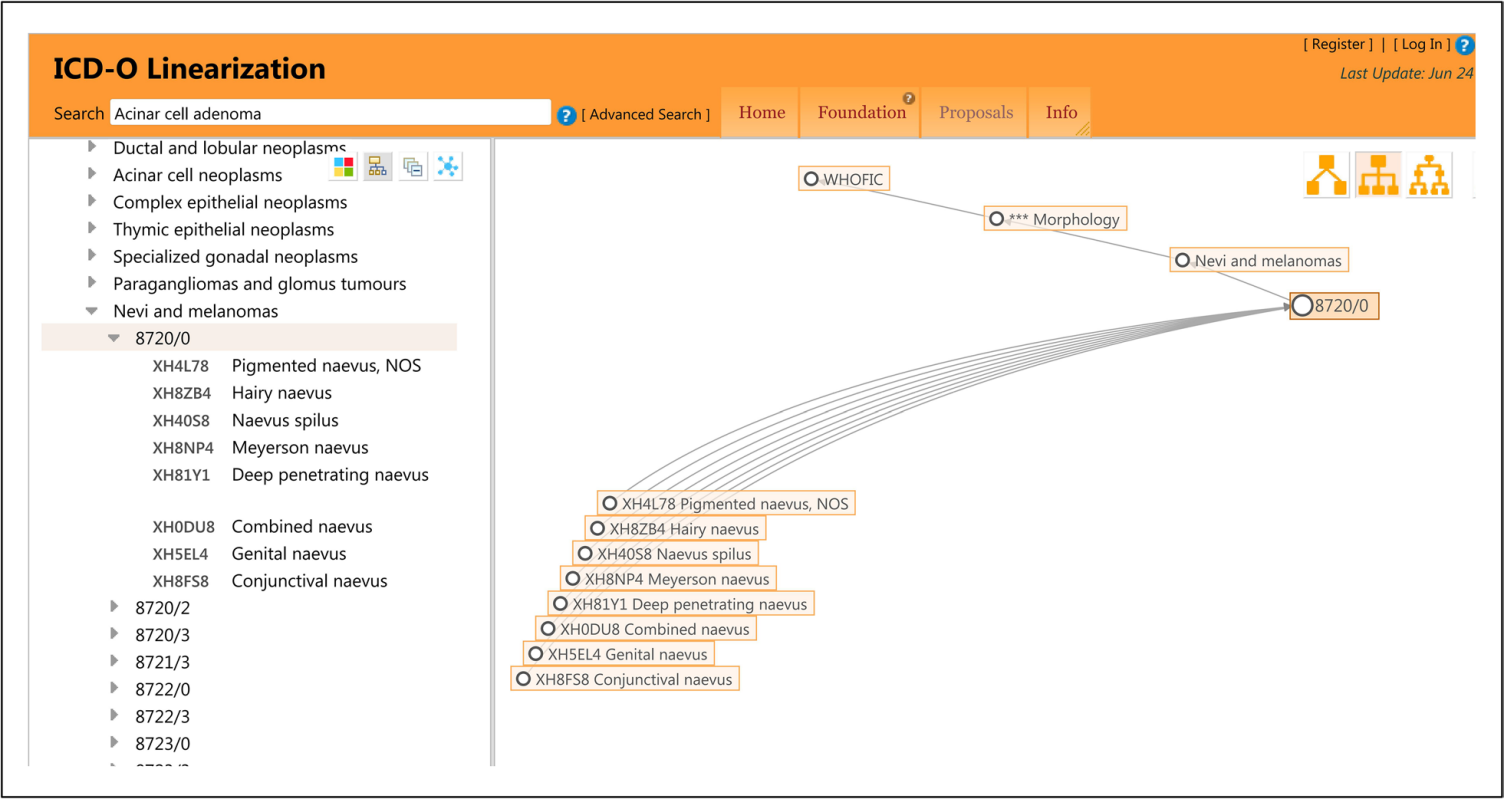
An example of the mutual compatibility of the morphology classification between the ICD-11 and ICD-O (2022/6/25)

In addition, the histopathology codes of the ICD-11 are compatible with the ICD-O linearization, as shown in Fig. 2. The most detailed category terms of the ICD-11 listed under 8720/0 are exactly the same as those of the ICD-O-3.2. Through the ICD-O linearization website, we can query the behaviour of the histopathology and compensate for the defect in the ICD-11, wherein the histopathology code cannot represent the behaviour.

The ICD-11 also has tremendous forwards compatibility. The WHO has established two groups (Medical and Scientific Advisory Committee, Classification and Statistics Advisory Committee) to maintain and update the ICD-11, and the process is meant to be open and transparent [2]. The entities of the Foundation Component could be continuously expanded in the process of maintenance and updating. During the revision process of the ICD-11, the entities regarding histopathology could also be maintained and updated according to the ICD-O.

Clearly, the rich Foundation Component, linearization derived from the Foundation Component and updating mechanism all support the compatibility of the ICD-11 with other classification systems. Good compatibility ensures an easy transition to the ICD-11, prevents the loss of critical data, and ensures its multiple use cases regarding neoplasms, such as certification and reporting of causes of death, Mortality and Morbidity Statistics (e.g., the burden of neoplasms), diagnosis-related grouping (DRG) (e.g., Medicare payment), quality and patient safety (e.g., adverse drug reactions), and cancer registries.

## High-level intelligence

The WHO provides an online browser and coding tool (‘blue browser’) for potential users in multiple languages; hence, compared to the ICD-O, the ICD-11 is entirely digital. The WHO also provides a maintenance platform (‘orange browser’), the content of which updates daily according to the user's proposals.

**Fig. 3:**
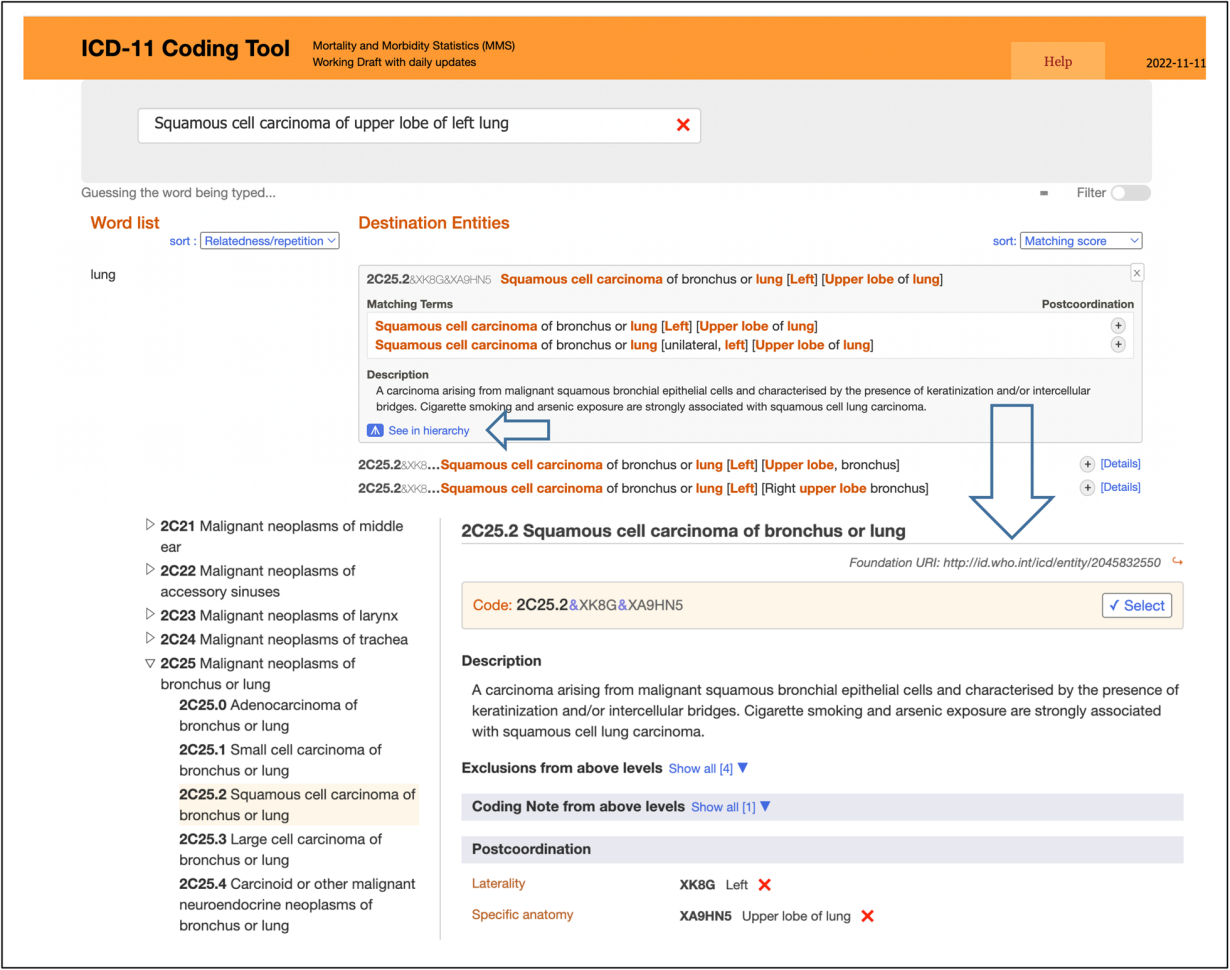
An example of smart coding with the ICD-11 coding tool (2022/11/12)

The ICD-11 smart coding tool [15] can guide coders to match appropriate stem codes according to the clinical diagnosis and provide postcoordination, replacing the previous use of the index volume of the ICD-10 in a paper environment [16]. The coding tool employs partial word matching, synonym management and flexible searching and is a simple automated method for potential users. When the search term corresponds to a cluster code instead of a single stem code, the tool can return the assembled cluster due to the virtual index, as shown in Fig. 3.

In addition, sanctioning rules are embedded in the coding tool and can verify whether the cluster code complies with the coding rules as plausibility checks. For example, when postcoordinating to form a cluster code, stem codes are always coded before extension codes. Moreover, the contents of the clinical description, exclusion, and coding notes are reflected in the browser to help with the coding checks. The powerful coding ability of the ICD-11 coding tool has been demonstrated in many field tests [17, 18].

In particular, the ICD-11 can provide ICD-11 MMS codes and uniform resource identifiers (URIs). The former are used for statistics, and the latter are a string of characters that can uniquely identify every entity and enable many of the capabilities of the ICD-11. When using URIs, the ICD-11 can provide more detailed concepts that far exceed the level of statistical categories, especially making rare diseases visible, which sometimes appear only in the Foundation Component and are below the shoreline of the ICD-11 MMS [19, 20]. As a classification system, URIs are a strong and beneficial supplement to the statistical function of the ICD-11 MMS codes.

## Conclusion

In general, the ICD-11 represents a novel classification system for neoplasm coding. It breaks the tradition of separate coding of topography and morphology, adds histopathology to the stem codes, distinguishes histopathology and behaviour via the stem codes, and obtains a meaningful minimum amount of information through the stem codes for statistics. As a multiaxial coding system, the optional usable extension codes of the ICD-11 allow us to refine the primary documentation of Chapter II of the classification using extension codes for detailed anatomy, histopathology, staging and grading information to support the usage of the ICD-O morphological categories, capture the clinical details and improve the coding granularity. In addition, the rich Foundation Component, linearization derived from the Foundation Component and updating mechanism all support the compatibility of the ICD-11 with other classification systems. Unlike previous classification systems, the WHO provides terminology coding with a smart coding tool, and coding in the ICD-11 can draw on statistical codes and URIs simultaneously. These features enable the capabilities of the ICD-11 to be more powerful for neoplasm coding than the ICD-O and basically meet the needs of stakeholders.

## Data Availability

The ICD-11 can be accessed at icd.who.int. The ICD-O can be accessed at www.iarc.fr/.

## Abbreviations

WHO: World Health Organization
ICD: International Classification of Diseases
ICD-9: International Classification of Diseases, 9th revision
ICD-10: International Classification of Diseases, 10th revision
ICD-11: International Classification of Diseases, 11th revision
ICD-O: International Classification of Diseases for Oncology
ICD-O-1: International Classification of Diseases for Oncology, First Edition
ICD-O-2: International Classification of Diseases for Oncology, Second Edition
ICD-O-3: International Classification of Diseases for Oncology, Third Edition
ICD-O-3.2: International Classification of Diseases for Oncology, Third Edition, Second Revision
IARC: The International Agency for Research on Cancer
IACR: The International Association of Cancer Registries
ICD-11 MMS: ICD-11 for Mortality and Morbidity Statistics
ICD-10-CM: International Classification of Diseases, 10th revision, clinical modification
NOS: Not otherwise specified
URIs: Uniform resource identifiers
DRG: Diagnosis-related grouping
